# Designing clinical-grade integrated strategies for the downstream processing of human mesenchymal stem cells

**DOI:** 10.1186/1753-6561-7-S6-P103

**Published:** 2013-12-04

**Authors:** Bárbara Cunha, Margarida Serra, Cristina Peixoto, Marta Silva, Manuel Carrondo, Paula Alves

**Affiliations:** 1 Instituto de Tecnologia Química e Biológica, Universidade Nova de Lisboa, Av. da República, 2780-157 Oeiras, Portugal; 2 iBET, Instituto de Biologia Experimental e Tecnológica, Apartado 12, 2780-901 Oeiras, Portugal; 3 Departamento de Química, Faculdade de Ciências e Tecnologia, Universidade Nova de Lisboa, 2829-516 Monte da Caparica, Portugal

## Background

During the past decade, human stem cells have been the focus of an increased interest due to their potential in clinical applications, as a therapeutic alternative for several diseases. Within this context, human mesenchymal stem cells (hMSCs) have gained special attention due to their immune-modulatory characteristics, as well as in secreting bioactive molecules with anti-inflammatory and regenerative features [1].

In order to face the high demands of hMSCs (from 10^5 ^to 10^9 ^cells per patient) [2] to be used in therapies, the establishment of robust manufacturing platforms that can ensure the efficient production, purification and formulation of stem cell-based products is still a challenge. Although substantial efforts have been performed on the development of clinical-grade bioprocesses for the expansion of hMSCs in microcarrier-based stirred culture systems, the incorporation of downstream strategies that assure efficient cell-bead separation and consequent hMSC concentration (i.e. volume reduction) and washing is required to deliver safe hMSCs to the clinic [3,4].

Therefore, the main aim of this work was the design of integrated methodologies (filtration and membrane technology approaches) [5] for the robust and clinical-grade downstream processing of hMSC.

## Materials and methods

Cell culture: hMSCs (STEMCELL Technologies™) were cultivated in IMDM supplemented with 10% of fetal bovine serum (FBS) or in MesenCult^®^-XF Medium (STEMCELL Technologies™) supplemented with 2 mM L-glutamine (Life Sciences) at 37°C in a humidified atmosphere of 5% CO_2_, according to manufacture recommendations. These cells were routinely propagated in static conditions (T-flasks) or on microcarriers (SoloHill Engineering, Inc) using stirred culture systems (spinner vessels and bioreactors). Cell concentration and viability were determined by counting the cells in a hemacytometer using the standard trypan blue exclusion method.

Cell characterization and quality control tests: Standard procedures for the analysis of cell surface markers (CD90, CD73, CD45, CD34) using flow cytometry tools, as well as cell-based assays for the evaluation of cell proliferation capacity (CFU assay - colony-forming unit) and differentiation potential (differentiation into osteoblasts and adipocytes) were performed, following the manufacturer's recommendations.

Downstream processing: After harvesting, the microcarriers were removed from the cell suspension using nylon filters (Millipore) with different pore sizes (100, 80 and 30 μm). The clarified cell-based materials were concentrated by tangential flow filtration (TFF) using polysulfone hollow-fiber cartridges with 0.45 μm pore size.

## Results

**Figure 1:**
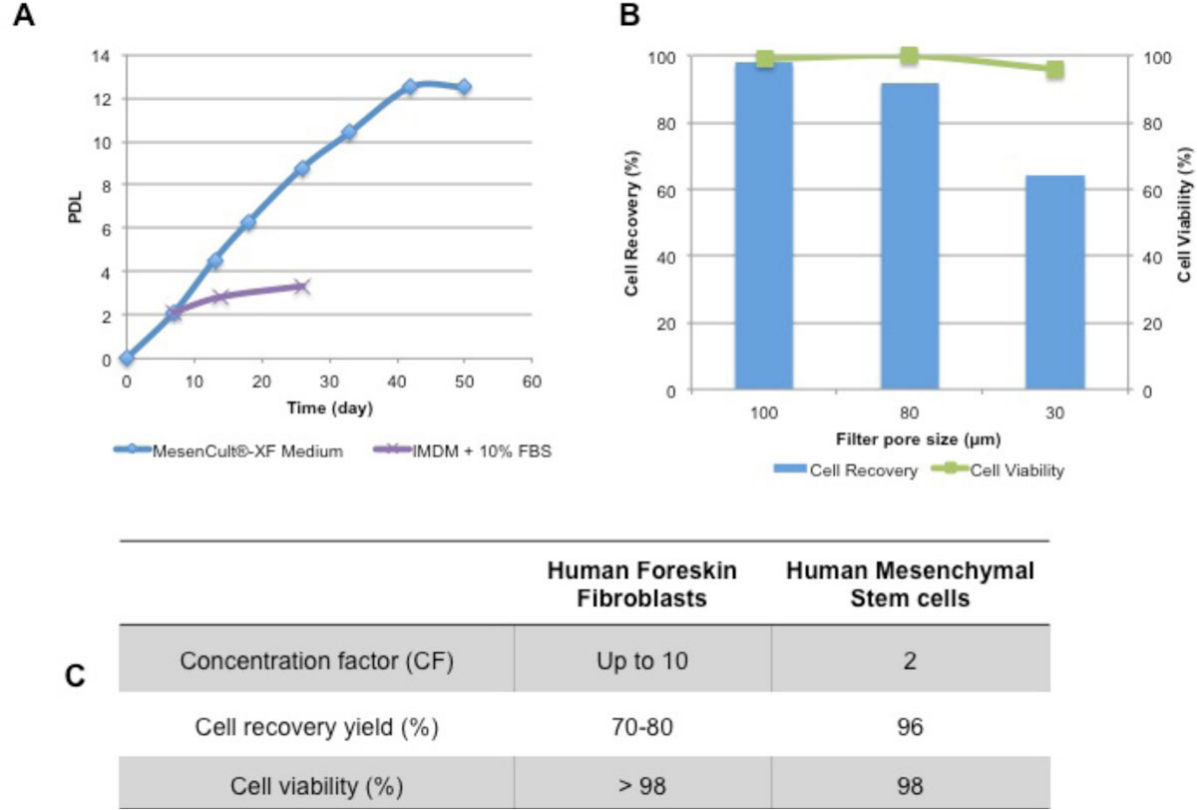
**Up- and down- stream processing of hMSCs**. **A) **Growth profile of hMSC culture; profile of cumulative PDLs of hMSC cultured in MesenCult^®^-XF (blue line) or in IMDM + 10% FBS (purple line) medium along culture time. **B) **Microcarriers' removal using nylon filters with different pore sizes (100, 80 and 30 μm). The (blue) bars represent the cell recovery yields, while the (green) line represents cell viability. **C) **Major outcomes achieved after TFF processing of human foreskin fibroblasts and hMSCs.

Over the past years, as scale-up platforms for the biomanufacturing of hMSCs become robust enough to yield high cell quantities to support cell-based therapies, culture media supplemented with FBS are becoming less used. This requirement is in line with what is advised by regulatory agencies, due to the main drawbacks associated to the use of FBS, such as the variability between different lots and suppliers and the risk of contamination with animal pathogens, which may trigger an immune response upon MSC therapy [5]. Within this context, large efforts have been made towards the development of serum- and xeno- free culture medium formulations for the expansion of hMSC. Thus, on a first approach, we evaluated the feasibility of propagating hMSCs in a serum- and xeno-free culture medium, the MesenCult^®^-XF medium, and further compared cell growth profile with standard medium formulation (e.g. IMDM + 10% FBS). Our results showed that, hMSC can be successfully expanded in MesenCult^®^-XF medium, presenting a constant population doubling length (PDL) of approximately 2 in each cell passage and a cumulative PDL of 12.5 in a total of 42 days (Figure 1A). Moreover, hMSCs showed an accelerated cell growth and increased lifespan when compared to the hMSC cultivated in standard culture medium supplemented with FBS where hMSCs presented limited proliferation capacity (Figure 1A). This was an expected outcome since MesenCult^®^-XF medium was design to enhance hMSC expansion from primary human bone marrow and cultured-expanded cells, leading to long-term cultures. It is important to mention that hMSCs maintained their characteristics after expansion in MesenCult^®^-XF medium, namely immunophenotype, proliferation capacity and multipotency (results not shown).

After the expansion of hMSCs in microcarrier-based stirred culture systems, different downstream strategies were evaluated for the purification of hMSCs. First, the clarification step was carried out to remove the microcarriers from the cell suspension. For this purpose, nylon filters were used and the effect of the mesh pore size on cell recovery yields and viability was evaluated. Our results showed that nylon is a suitable material for the clarification step since it ensured efficient removal of microcarriers (no beads were detected after filtration processing) without compromising cell viability (Figure 1B). Moreover, we demonstrated that higher mesh pore sizes yielded higher cell recoveries (Figure 1B).

For the cell concentration and volume reduction step, preliminary experiments were performed with human foreskin fibroblasts (Figure 1C). With this cellular system, a concentration factor (in volume) of 10 times was successfully achieved using TFF processes, yielding 70-80% of recovered cells with high viabilities (Figure 1C). Process validation with hMSCs is ongoing but first results were encouraging since we were able to concentrate 2 times hMSCs while ensuring high cell recovery yields (96%) and viabilities (98%) (Figure 1C). In addition, hMSCs maintained their immunophenotype, as well as their proliferation capacity and multilineage differentiation potential at the end of all steps of the downstream process (results not shown).

## Conclusions

While upstream technologies mature to meet the increasing demand of hMSCs, biomanufacturing bottlenecks are now shifting towards the downstream processing of stem cells. This work shows our first approach to tackle such bottlenecks. More specifically, we demonstrate that standard filtration techniques and TFF systems are suitable and robust approaches for the downstream processing of hMSCs. Using these strategies we were able to ensure efficient removal of the major impurities of the cellular suspension (microcarriers) and further concentrate cell-based products up to 10 times without compromising their viability and quality. However, further improvements in cell concentration and polishing steps are still required. Nonetheless, this work provides important insights towards the establishment of robust and clinical-grade bioprocesses for the purification of hMSCs to be integrated and applied in the biomanufacturing of cell-based therapies.
